# Endemic Typhoid

**Published:** 1897-12-11

**Authors:** 


### Endemic Typhoid.

The experimental investigations as to the etiology of endemic typhoid described by Sir Richard Thorne at the last meeting of the Royal Medical and Chirurgical Society are of great interest and importance, showing, as they do, how extensively the typhoid organism may grow and how long it may persist when once implanted in organically polluted soil. These investigations explain much that has hitherto been a matter of guess work, and express in accurate terms of bacteriology a great deal that has so far been a matter of faith rather than of understanding.

Popular interest in typhoid fever seems to hinge almost entirely on its epidemics. There is something dramatic and sensational in a city being struck as in a night by a disease which in a short time lays low almost a tenth of its inhabitants. Still, all this is a mere matter of mechanical distribution. What is of real scientific interest is the question, Where does the microbe live; where does it maintain its existence month after month and year after year; and whence does it come when it causes the sporadic outbreaks—a case here and a case there dotted over the country—by which the disease shows itself when its distribution is not assisted by such modern appliances as water-pipes and drains?

It used to be maintained that typhoid fever sprang from foul emanations, and that it could be generated anew by decomposition of sewage and animal filth. This view was ably maintained by the late Dr. Murchison, and would, no doubt, have held its own but for the striking facts afforded by various water epidemics, which showed how strict was the relationship between the spread of the disease and the specific infection of the water by some pre-existing case. The fact is, that when it was once proved that the infective material was a specific germ the notion that mere filthy surroundings were capable of setting up the disease seemed to be knocked on the head. Some source of infection was, therefore, looked for in each group of cases. In great epidemics this was often not very difficult of discovery by skilled investigators; but in sporadic country outbreaks the most careful search for the source of the infection has again and again proved fruitless, and thus the doctrine of the pythogenic or filth origin of typhoid fever has never been entirely without followers among clinical observers. Thus, curiously enough, it happened that this fever, which in the outbreaks connected with water and milk supply has furnished some of the very best examples of a zymotic or germ-spread malady, has also in regard to certain country outbreaks remained the greatest stumbling-block in the way of the general acceptance of the germ theory of infectious diseases.

What Sir Richard Thorne told to the meeting, however, was this: That as the result of experiments undertaken on behalf of the Medical Department of the Local Government Board by Dr. Sidney Martin, it had been found that when organically polluted soil, derived from localities where typhoid fever was wont to recrudesce from time to time, was inoculated with the typhoid bacillus, this organism not only retained its vitality, but grew, and in some cases permeated the whole mass of the soil experimented on, and that in such soils the typhoid bacillus had retained its power of growth for so long a period as nine months. There was, in fact, no reason to believe that the limit of its vitality in such soils had yet been nearly reached. When, however, it was grown under similar laboratory conditions in virgin soils collected at a distance from human habitation no trace whatever could be found later than 23 days after the inoculation. Such soil, in fact, was found to be quickly destructive to the organism. We seem here to see an explanation of the origin of endemic typhoid which fits in with all the facts. When typhoid is imported into a place with clean surroundings, and a clean soil, however virulent it may be, it soon dies out. Where, on the other hand, the soil is polluted with decomposing organic, and especially animal matter, the typhoid organism takes root and not only grows but persists, so that whenever the temperature rises to the proper heat and the soil is moistened to the proper degree a great development of the typhoid bacilli takes place, and these, washed into wells or streams from which drinking water is drawn, again gain access to man and thus at last give rise to cases of typhoid fever, the origin of which no one can trace, so long is it since the infection was planted in the soil. This is the view that has been held by many as a theory for some time, and it is extremely satisfactory to find it working out true, as we have said, in terms of bacteriology when it is put to the experimental test in the laboratory, for it explains much.

